# Coping strategies in challenging situations among informal caregivers: validation of the newly developed six-item German short version of the Brief COPE Inventory (COPE 6)

**DOI:** 10.1186/s40359-025-03815-5

**Published:** 2025-12-13

**Authors:** Natascha Lauer, Elmar Graessel, Paula Hinkl, Nicolas Rohleder, Anna Pendergrass

**Affiliations:** 1 Center for Health Services Research in Medicine, Department of Psychiatry and Psychotherapy, Uniklinikum Erlangen, Friedrich-Alexander-Universität Erlangen-Nürnberg (FAU), Schwabachanlage 6, Erlangen, 91054 Germany; 2 Chair of Health Psychology, Department of Psychology, Friedrich-Alexander-Universität Erlangen-Nürnberg, Erlangen, Germany

**Keywords:** COPE 6, Brief COPE, Caregivers, Coping strategies, Item difficulty, Discriminatory power, Principal component analysis, Reliability, Validity

## Abstract

**Background:**

The Brief Coping Orientation to Problems Experienced Inventory (Brief COPE) is a well-established instrument for assessing coping strategies. Nevertheless, research has shown that it has a heterogenous factor structure, thus suggesting the need for a more economical version that considers previously identified broader factor structures. Consequently, the COPE 6 was developed as a six-item short version that measures functional coping (FC) and dysfunctional coping (DC) strategies. The aim of this study was to validate the COPE 6.

**Methods:**

The validation sample included 961 German informal caregivers. A principal component analysis was conducted. Cronbach’s alpha and the Spearman-Brown formula were calculated to analyze reliability. The item characteristics were identified by discriminatory power and item difficulty indices. Construct validity was analyzed by testing five hypotheses.

**Results:**

Two components were extracted: FC and DC. The reliability was α = .71 for the FC subscale and *r*_*sb*_ = .16 for the DC subscale. The FC subscore was largely associated with the Brief COPE FC subscore and weakly associated with the Benefits of Being a Caregiver Scale score. The DC subscore showed small correlations with both the Brief COPE DC subscore and the short version of the Burden Scale for Family Caregivers score. Correlations with demographic factors and the subscales of the Social Desirability-Gamma Short Scale were negligible to small.

**Conclusion:**

The COPE 6 is the first theory-based adaptation of the Brief COPE to measure FC in an economical way. The FC subscale represents a valid and reliable tool for assessing FC strategies. The current form of the DC subscale appears to measure two extreme aspects of DC with insufficient convergent validity so far. Additional research is needed to develop this subscale further.

**Supplementary Information:**

The online version contains supplementary material available at 10.1186/s40359-025-03815-5.

## Background

### Research on coping strategies among informal caregivers

Coping is one of the most frequently studied constructs in psychological research [1, 2]. In general, coping strategies can be understood as any mechanisms (actions, behaviors, or thoughts) that help people deal with internal or external stresses and challenges [3, 4]. Coping strategies have been studied in a number of populations [5–10] but are currently being studied more frequently in informal caregivers [11, 12]. One reason for the growing interest in informal caregivers in coping research is their vital importance in healthcare systems, particularly given the increasing demand for care due to ageing populations worldwide [13–15]. In Germany, more than 4.1 million care receivers are cared for at home, 2.5 million of them exclusively by informal caregivers [16]. Most informal caregivers are affected by consistently elevated stress levels, and those who care for older care receivers face elevated burden [17, 18]. Nevertheless, despite the fact that the majority of informal caregivers are affected by negative consequences, they also experience positive aspects of caregiving at the same time [19]. Even though these benefits have received less attention thus far [15], just the fact that they exist suggests that there may be coping strategies that help informal caregivers manage the burdens of the care situation. Gräßel and Adabbo [20] noted that informal caregivers’ coping strategies reflect how they manage a stressful homecare situation. Depending on the type of coping strategy that is used, both positive (e.g., benefits) and negative (e.g., burden) effects can ensue. Studies have found associations between functional coping (FC) strategies and better adaptation to stress as well as between dysfunctional coping (DC) strategies and increased perceptions of stress [21], and, consequently, negative psychological outcomes [22].

### Conceptual and measurement challenges of the coping construct

Despite the large body of research, taxonomies and measurements of coping have varied widely [1, 2, 23–25]. In the transactional model of stress and coping [26], Lazarus and Folkman distinguished between two broad categories of coping: emotion-focused and problem-focused coping strategies [27]. This approach is most commonly used in coping research and has dominated the development of other coping theories as well as multiple coping instruments [1, 23, 25, 28]. This macroanalytic dichotomization of coping has often been criticized for oversimplifying coping and blurring distinctions between categories [24, 29, 30].

Consequently, Carver et al. [30] enhanced this coping concept by integrating aspects of their model of behavioral self-regulation [31]. Besides the more useful (functional) problem-focused and emotion-focused coping categories, they extended the coping taxonomy by incorporating less useful (dysfunctional) coping strategies into it [23, 30, 32]. According to Frydenberg [1], functional styles include “direct attempts to deal with the problem, with or without reference to others,” whereas the dysfunctional styles can be described as “non-productive strategies” (p. 87). In recent studies, the existence of two (functional and dysfunctional) or three (problem-focused, emotion-focused, and dysfunctional) theory-based broad dimensions of coping have been discussed [8, 11, 22, 28, 30, 33–37].

Due to the varying conceptualizations of the construct, numerous different measurement tools have been developed to operationalize coping strategies [38–40]. This heterogeneity in the operationalization of coping has led to different and sometimes incomplete aspects of coping being measured or, in some cases, entirely distinct constructs [41]. Moreover, some researchers have voiced the criticism that numerous extensive instruments have emerged empirically without a theoretical foundation [30, 32]. Based on the Coping Orientation to Problems Experienced Inventory [30], the abbreviated Brief Coping Orientation to Problems Experienced Inventory (Brief COPE) [32] was developed. The Brief COPE measures 14 coping dimensions with a total of 28 items. It is based on a microanalytical coping approach and represents the coping construct in a comprehensive and theory-based way [28]. The Brief COPE has been translated into multiple languages [e.g., 42–48] and has been frequently applied in various populations [e.g., 11, 22, 28, 33, 49–55].

### Heterogeneity in the factor structure of the Brief COPE

Despite its widespread dissemination and multiple validations, the factor structure of the Brief COPE remains disputed [3, 28, 56, 57]. Solberg et al. [28] showed that the original 14-factor structure has rarely been replicated in past research. The number of coping dimensions identified in 85 research articles varied from two to fifteen factors (median 8.50), with a dichotomous factor structure being the most frequently identified [28]. However, attempts to robustly identify broader coping categories have shown ambiguous results as well [56, 58]. Results have depended on multiple factors of influence, such as sample characteristics, contextual factors, and cultural factors [58, 59]. For example, when limited to specific populations (e.g., informal caregivers) [11], past results on a higher order structure have been more homogeneous. Likewise, the version of the Brief COPE that is used (situational vs. dispositional coping) seems to influence how the coping construct is represented [28, 60].

According to Solberg et al. [28], organizing the Brief COPE items into more essential broader coping dimensions enables a beneficial examination of differential or predictive validity. Consequently, it was suggested that more research is needed to evaluate shorter versions of the Brief COPE in order to obtain more practically relevant instruments.

### Study aims

To overcome past limitations and adopt recent suggestions [28], our overall aims were to create and validate the COPE 6 as an instrument based on the Brief COPE that offers high test economy without comprising validity or reliability, and that yields a stable factor structure. Our objectives concerning the construction of the COPE 6 were derived as follows: (1) The first aim was to improve feasibility by developing an abbreviated form of the Brief COPE that measures coping strategies in a time-efficient way, suitable for academic research and caregiving contexts. (2) The second aim was to minimize item redundancy and to assess the coping construct as concisely as possible. (3) The third aim was to enhance item clarity by including only precisely worded items (see section “COPE 6 – scale development and description, third step”). Furthermore, we aimed to validate the COPE 6 by examining (1) its underlying component structure, (2) item characteristics (discriminatory power, item difficulty), (3) reliability, and (4) convergent and discriminant validity.

## Methods

### Study design

The cross-sectional data for the validation of the COPE 6 were obtained from the “Benefits of Being a Caregiver” study, a large representative survey of informal caregivers in Bavaria (Germany) between October 2019 and March 2020. The second survey, conducted to generate the subsample and obtain missing variables necessary for a comprehensive examination of construct validity, was carried out between September 2022 and January 2023.

### Procedure

A total of 50 care assessors from the Medical Service of the Bavarian Health Insurance distributed 5,000 questionnaires to statutorily insured informal caregivers who applied for an initial grading or an increase in their care receivers’ care level at the Medical Service of the Bavarian Health Insurance. Participants completed the questionnaires in paper-and-pencil format and sent them back by mail. Participation in the study was entirely voluntary. A total of 1,084 (21.7%) of the distributed questionnaires were returned. The validation sample comprised *N* = 961 informal caregivers, after excluding those caring for care receivers younger than 65 years.

The primary research question of the “Benefits of Being a Caregiver” study focused on examining the concept of “experienced benefits”. Consequently, two instruments necessary for a complete evaluation of the construct validity of the COPE 6 (Brief COPE, Social Desirability-Gamma Short Scale) were not included in order to ensure that the size of the questionnaire was manageable. Therefore, a subsample of *n* = 176 informal caregivers from the initial sample that continued to care for their relatives and had given consent to being contacted again was surveyed a second time using the Brief COPE, the Social Desirability-Gamma Short Scale, and the COPE 6. Participants completed the questionnaires in paper-and-pencil format and sent them back by mail. A total of 106 (60.2%) of the distributed questionnaires were returned. The final subsample consisted of *n* = 81 informal caregivers excluding individuals younger than 65 years, as well as those with more than 50% missing data or inconsistent response patterns in the questionnaire. For a detailed illustration of the sample composition, see Supplementary Figure S1. Approval was obtained from the ethics committee of the Medical Faculty of the Friedrich-Alexander-Universität Erlangen-Nürnberg (No.: 220_20 B).

### Participants

To qualify for inclusion, participants had to be residents of Bavaria, fluent in German, covered by statutory health insurance, and had to be the main informal caregiver of the care receiver. Furthermore, the informal caregivers had to have applied for an initial assessment or an upgrade in the level of care by the Medical Service of the Bavarian Health Insurance for which they were eligible. For both samples, analyses were limited to informal caregivers who cared for elderly individuals (> 64 years) to approximate the permanent burden of a gerontological care situation in a standardized way. Informal caregivers of care receivers younger than 65 years were excluded from the study. There were no restrictions on the ages of the informal caregivers themselves.

### Sample characteristics

After excluding 121 cases because the care receivers were younger than 65 years (see Supplementary Figure S1), the validation sample comprised 961 informal caregivers. Informal caregivers had a mean age of 62.10 years (*SD* = 12.63), and most were women (75.7%). A total of 61.0% were children (or children in law), 30.5% were spouses, and 8.5% were other (non)relatives (e.g., aunts, nephews, friends). The sample of informal caregivers showed a high level of objective burden with 8.85 h of care per day on average. A total of 47.8% of the informal caregivers were employed in addition to providing informal care. The care receivers were on average 82.12 years old (*SD* = 7.04), and most of them were female (66.9%). A total of 52.8% of the informal caregivers lived with their relatives. Table 1 presents a comprehensive overview of all sample characteristics. For more information about the subsample, see Supplementary Table S1.

**Table 1:** Sample characteristics (*N* = 961)

**Characteristics** ^**a**^	***n *** **(%)**	***M*** (*SD*)
**Informal** **Caregiver**		
Age (years)		62.10 (12.63)
Gender (female)	727 (75.7)	
Highest education level		
No school-leaving qualification	5 (0.5)	
Lower secondary school	365 (38.0)	
Middle school	408 (42.5)	
Advanced school-leaving examination	88 (9.1)	
University degree	95 (9.9)	
Gainfully employed (yes)	459 (47.8)	
Duration of care (months)		48.45 (78.72)
Relationship		
Spouse	293 (30.5)	
Parents (in law)	586 (61.0)	
Others	82 (8.5)	
**Care** **Receiver**		
Age (years)		82.12 (7.04)
Gender (female)	643 (66.9)	
Dementia (yes)^b^	364 (37.9)	
Level of care (1–5)^c^	768 (79.9)	
**Care** **Situation**		
Living together (yes)	507 (52.8)	
More than one care receiver (yes)	59 (6.1)	
ADL (h/d)^d^		2.69 (2.24)
IADL (h/d)^d^		3.45 (2.25)
Supervision (h/d)^d^		2.70 (3.25)
Hours of care (total)^e^		8.85 (5.12)
Informal help received (yes)^f^	576 (59.9)	

*n*/*N *sample size, *M *mean, *SD *standard deviation, *h/d *hours per day

^a^Validation sample (*N* = 961 informal caregivers from the “Benefits of Being a Caregiver” study)

^b^Dementia as the (main) reason care is needed

^c^Describes the dimension of care needs assessed by trained experts (independent of the insurance system) that represents the basis for approving formal care services financed by long-term care insurance, Range 0 (a little care needed) to 5 (a lot of care needed)

^d^h/d spent on activities of daily living (ADL), instrumental activities of daily living (IADL), and supervision of the care receivers (Range 0–17)

^e^Total care time per day (Range 0–17) including ADL (h/d), IADL (h/d), and supervision (h/d)

^f^Support from friends, relatives, or acquaintances

### Instruments

All instruments were presented in German.

#### COPE 6 – scale development and description

The development of the COPE 6 was guided by the main goal of finding the most valid and economical way to assess coping strategies. The process of scale development was characterized by a deductive, theory-based [1, 11, 23, 26–28, 30, 32, 61, 62], multi-step approach conducted by experts who are researchers in psychological and medical contexts. Current guidelines for scale construction were taken into account [63].

First, a literature search was conducted to obtain an overview of existing quantitative coping questionnaires. The identified instruments were evaluated according to the degree of establishment, validation quality, and the degree of conceptual clarity of the coping construct. The Brief COPE [32, 43] was finally selected based on quality criteria. This theory-based questionnaire offers the opportunity to measure the construct of coping in a highly differentiated way [28] and to evaluate dispositional or situational coping strategies [32, 43].

In a second step, the 28 Brief COPE items were literature-based screened for their categorization into the three dimensions of coping strategies: functional problem-focused, functional emotion-focused dimensions, and dysfunctional. Our approach was strongly based on Carver´s concept underlying the construction of both the original Coping Orientation to Problems Experienced Inventory [30] and the abbreviated Brief COPE [32]. This classification was chosen because it is one of the most established and thoroughly researched theoretical framework for coping strategies [23, 27, 61]. Therefore, despite of the significant reduction in the number of items, a wide range of aspects of the coping construct can still be assessed, remaining as close as possible to the original of the Brief COPE.

In a third step, the specific COPE 6 items were selected. The final item selection was based on a two-step exclusion procedure conducted in a focus group (a medical doctor, one psychologist with special expertise in psychometry and two former informal caregivers) following expert consensus and current guidelines for scale construction [63]. Excluded were (a) redundantly formulated items (e.g., “I’ve been using alcohol or other drugs to help me get through it” and “I’ve been using alcohol or other drugs to make myself feel better”) and (b) items with insufficient or ambiguous discriminative power (e.g., “I’ve been praying or meditating”). Additionally, for inclusion two criteria had to be fulfilled: (a) items had to assess the coping dimensions problem-focused, emotion-focused and dysfunctional as comprehensively as possible and (b) closely fitting the target population [64]. The result was a total of six items comprising the final COPE 6. Based on the underlying theoretical framework used for scale development [27, 30, 32, 61], these included two items representing emotion-focused coping, two items representing problem-focused coping, and two items representing DC.

**Fig. 1:**
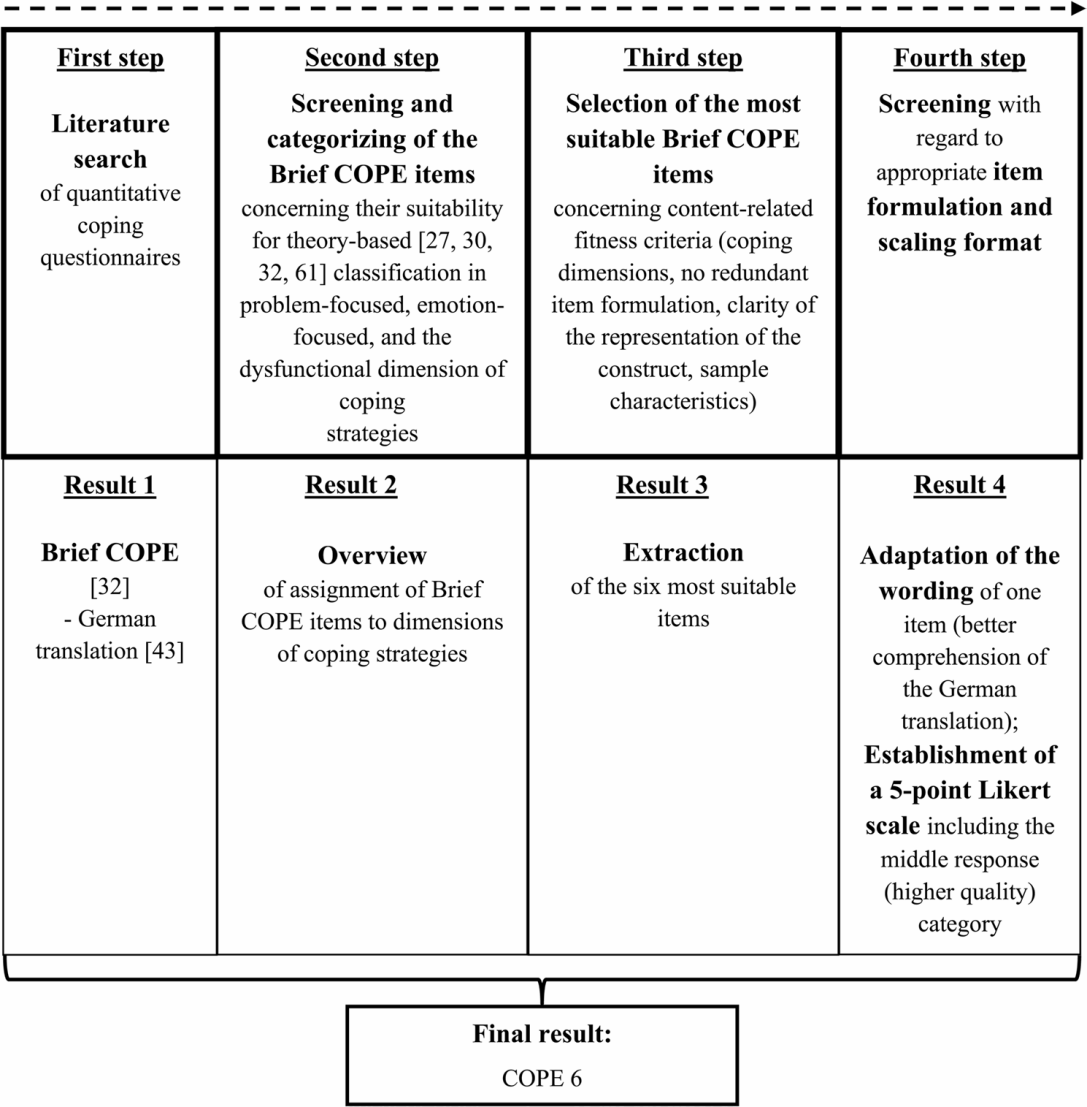
Process followed to develop the COPE 6

In a final fourth step, the included items were reviewed regarding appropriate wording and response format. To ensure content validity through linguistic clarity [64] in the German version, the wording of one item had to be revised (original item “Ich habe aufmunternde Unterstützung von anderen erhalten” was adapted to “Ich habe Aufmunterung von anderen erhalten”). To improve data quality, a 5-point Likert scale with a central response option was implemented [65]; ranging from 0 (strongly disagree) to 4 (strongly agree). For a brief overview of the scale development process, see Fig. 1.

Higher sum scores on the FC (Range 0–16) and DC subscales (Range 0–8) describe more frequent use of the corresponding coping strategies. To assess dispositional coping, the instruction was formulated without reference to specific situations: “The following statements refer to your thoughts and actions. How did you behave in past unpleasant or difficult situations?” [43, 44]. The final version of the COPE 6 can be found in Appendix A (English version) and Appendix B (German version).

#### Brief COPE

Coping strategies were measured with the well-established German version [43, 44] of the Brief COPE [32]. It is a 28-item self-report questionnaire consisting of 14 subscales comprising two items each. Carver [32] grouped these 14 subscales into three dimensions: more functional *problem-focused coping* and *emotion-focused coping* and more *dysfunctional coping*. Based on the literature [8, 30, 34–37, 66–68], the total composite scores for FC (including the *emotion-focused coping* and *problem-focused coping* subscales) and DC (including the *dysfunctional coping* subscale) were built. Due to a bias in the German translation of the item “I’ve been giving up the attempt to cope” which represents a more *active coping* strategy and is therefore associated with *problem-focused coping*, we decided to include this item in the FC subscale for reasons of better fit. Dispositional coping strategies were assessed by formulating the instructions in a situation-independent way [43, 44]. Responses to all items were given on a 4-point scale ranging from 0 (I haven´t been doing this at all) to 3 (I´ve been doing this a lot). The sum scores ranged from 0 to 51 for the FC subscale and 0 to 33 for the DC subscale. Higher scores represent more frequent use of the corresponding coping strategy. Against the background of the heterogeneous factor structure reported in past studies [28], Cronbach’s alpha ranged from .81 to .88 for the FC subscale and from .48 to .81 for the DC subscale in previous research [8, 36, 68]. In this sample, Cronbach’s alpha was .77 for the FC subscale and .53 for the DC subscale, consistent with values reported in previous research.

#### Benefits of Being a Caregiver Scale

The Benefits of Being a Caregiver Scale is a validated instrument for measuring the benefits of being an informal caregiver [19]. The scale comprises 14 items. Participants rate items on a 5-point scale ranging from 0 (strongly disagree) to 4 (strongly agree). The total sum score ranges from 0 to 56. Higher scores indicate that the informal caregiver has experienced greater benefits. Cronbach´s alpha for the German version of the Benefits of Being a Caregiver Scale was previously .92 [19]. In this study, Cronbach´s alpha was .92.

#### Short version of the Burden Scale for Family Caregivers

Subjective burden in informal caregivers was measured with the short version of the Burden Scale for Family Caregivers [69], which consists of 10 items. Raters’ self-assessment occurs on a 4-point scale ranging from 0 (strongly disagree) to 3 (strongly agree). The total sum score ranges from 0 to 30. Higher degrees of agreement indicate higher subjective burden. Cronbach´s alpha for the German version of the short version of the Burden Scale for Family Caregivers was previously .92 [69]. In the present study, Cronbach´s alpha was .92.

#### Social Desirability-Gamma Short Scale

The Social Desirability-Gamma Short Scale is a well-validated instrument available in English [70] and German [71]. It measures two aspects of social desirability: *exaggerating positive qualities* and *minimizing negative qualities*. Each subscale consists of three items. Every item is rated on a scale ranging from 0 (doesn’t apply at all) to 4 (applies completely). Internal consistency was calculated with McDonald´s omega. The social desirability scale score is computed separately by calculating the unweighted mean score of the three items on each subscale (exaggerating positive qualities and minimizing negative qualities). Higher scores on *exaggerating positive qualities* represent a more pronounced socially desirable response pattern. On *minimizing negative qualities*, lower scores indicate a more pronounced socially desirable response pattern. McDonald´s omega for the German version of the questionnaire was previously .71 for the *exaggerating positive qualities* subscale and .78 for the *minimizing negative qualities* subscale [70]. In the present sample, McDonald´s omega was .61 for the *exaggerating positive qualities* subscale and .70 for the *minimizing negative qualities* subscale.

#### Other measures

Sociodemographic and background information was assessed to describe the sample characteristics. They included the informal caregiver’s age and gender, educational and employment status, duration of care in months, and the informal caregiver’s relationship to the care receiver (categorized into spouses, parents [in law], and others). Besides, the care receiver’s age and gender, the reason for care (categorized into dementia and non-dementia) and the level of care (5-level ordinal scale ranging from 0 = a little care needed to 5 = a lot of care needed) were collected. To describe the homecare situation, information was collected on the living situation (living together, yes or no), whether there is more than one care receiver to care for (yes or no), and information on informal care time (number of hours per day the informal caregiver provides care to the care receiver; Range 0–17). Informal care time was composed of the three aspects *Activities of daily living* (e.g., dressing, bathing, toilet visits), *Instrumental activities of daily living* (e.g., meal preparation, assistance with taking medication or housekeeping), and *Supervision* (e.g., avoiding dangerous situations), each evaluated with one item in accordance with the *Resource Utilization in Dementia questionnaire* [72]. Informal help was assessed with the single item “Do you currently receive help from relatives, friends, acquaintances with caregiving?” (yes or no).

### Statistical analyses

The majority of the calculations were performed with the IBM SPSS Statistics software version 28 for Windows. The calculation for the reliability of the Social Desirability-Gamma Short Scale (McDonald’s omega) was done with JASP statistical software. The significance level was set at α = .05.

To plausibly estimate missing values, imputation methods were used. For metric scales, total missing scores were imputed via the expectation–maximization algorithm. For metric scaled variables with no sum scores, the expectation–maximization algorithm was also used to impute single missing values. We conducted modus imputation of the categorial scaled variables (< 5.0% missing values).

#### Descriptive statistics

We computed descriptive statistics for characteristics of the informal caregivers, care receivers, and care situation. We used the mean (*M*) and standard deviation (*SD*) for continuous variables and absolute (*n*) and relative (%) frequencies for categorial variables. The mean, median, standard deviation, and skewness were calculated to describe the distributions of the FC and DC subscores.

#### Principal component analysis

Due to the large heterogeneity in the factor structure of the Brief COPE reported in many previous studies [28], a principal component analysis was conducted. On the basis of the identified underlying structure, we formulated hypotheses to subsequently test the convergent and discriminant validity and further assess the construct validity of the COPE 6. This structure-seeking procedure was based on Moosbrugger and Kelava’s specifications for testing construct validity [73]. The Kaiser-Meyer-Olkin measure of sampling adequacy with recommended values greater than .60 [74] and Bartlett´s test of sphericity with a recommended *p*-value less than .05 [74] were used to test the requirements. Orthogonal Varimax rotation was used to obtain a simple structure for the variable grouping. The scree test was used to show the distribution of the eigenvalues for the individual components. In the case of ambiguous scree test results, parallel analysis [75] was applied to determine the final number of components to be extracted. A component loading ≥ .50 was defined as the criterion for assigning a variable to a component [76].

#### Item analysis and reliability

To conduct the item analysis, we computed the mean and standard deviation, the difficulty index, and the discriminatory power at the item level. For item difficulty, a corridor of .20 to .80 is recommended [65]. For discriminatory power, values of .30 to .50 can be classified as moderate and a power of > .50 as high [65]. The scale reliability was examined via internal consistency and split-half reliability. Therefore, for identified components with more than two items, Cronbach’s alpha was evaluated, whereas for identified components with two items, the Spearman-Brown coefficient (*r*_*sb*_) was calculated [77]. The following criteria were used for Cronbach´s alpha: excellent (α > .90), good (α > .80), acceptable (α > .70), questionable (α > .60), poor (α > .50), and unacceptable (α < .50) [78]. The interpretation of the Spearman-Brown coefficient follows general reliability measure conventions with sufficient values ≥ .70 [79].

#### Convergent and discriminant validity

To examine the construct validity of the COPE 6, the following five hypotheses were tested. Hypotheses 1–3 reflect convergent validity, whereas hypotheses 4 and 5 represent discriminant validity.

##### Hypothesis 1

Because the COPE 6 represents an extra-short version of the Brief COPE, the two questionnaires were assumed to measure the same coping construct. Therefore, we hypothesized large positive correlations between the COPE 6 FC and DC subscale scores and the corresponding Brief COPE subscale scores [28].

##### Hypothesis 2

In previous research, perceived positive aspects of caregiving were described as mediators that buffer negative outcomes, such as caregiving burden [80] or perceived care-related (di)stress [81]. Thus, perceived benefits themselves are considered positive coping resources [82]. FC strategies have been shown to be correlated with perceived positive aspects of caregiving [83, 84]. Consequently, we hypothesized that there would be a positive correlation between the COPE 6 FC subscale score and the Benefits of Being a Caregiver Scale score.

##### Hypothesis 3

Research has shown that there is a positive association between informal caregivers’ subjectively perceived burden and DC strategies [20, 33, 52, 85–87]. Thus, an increased sense of burden should be associated with a greater application of DC strategies in informal caregivers. Therefore, we hypothesized that there would be a positive correlation between the short version of the Burden Scale for Family Caregivers score and the COPE 6 DC subscale score.

##### Hypothesis 4

In past research, no meaningful association between coping strategies and social desirability has been found [30, 88]. Thus, we hypothesized no meaningful correlation between either COPE 6 subscale score and the Social Desirability-Gamma Short Scale subscale scores.

##### Hypothesis 5

Past research has shown no meaningful correlations between sociodemographic factors and informal caregivers’ use of coping strategies [37, 87, 89]. Even though some primary studies reported incoherent associations [90], reviews have shown that there is a lack of clear overall evidence [91]. On the basis of this research, we hypothesized only an inconsequentially small correlation between the COPE 6 subscale scores and selected sociodemographic variables (age, gender, education level, and employment status).

To test hypothesis 1 to hypothesis 5, we computed correlations. We calculated Pearson´s correlation coefficient (*r*_*p*_) between the COPE 6 subscores and metric data, Spearman´s rank correlation coefficient (*r*_*s*_) between the COPE 6 subscores and ordinal data or if there was a violation of underlying assumptions for Pearson´s correlation analyses, and point biserial correlation coefficient (*r*_*pb*_) between the COPE 6 subscores and dichotomous data [65]. Correlations .50 or greater were classified as large effects, those between .30 and .49 as medium effects, and those between .10 and .29 as small effects. Correlations smaller than .10 indicated no association [65]. Due to the different sizes of the validation sample and the subsample, a comparative interpretation of the sample size-sensitive *p*-values was not possible. Therefore, effect sizes were primarily used to evaluate the construct validity. To prevent an accumulation of the alpha error, we applied the Benjamini-Hochberg method [92].

## Results

### Distribution of the COPE 6 subscores

The distribution of the COPE 6 FC subscore ranged from 0 to 16, representing all theoretically possible score values. The mean was 7.77, and the median was 8.00 (*SD* = 3.58). Scores were normally distributed with a skewness of −0.04 (Supplementary Figure S2). The distribution of the COPE 6 DC subscore ranged from 0 to 8, which corresponded to the entire theoretically possible scale range. The mean was 1.86 (*SD* = 1.55), and the median was 2.00. With a positive skewness of 0.80, the distribution was right-skewed (Supplementary Figure S2). Descriptive statistics for all scales and subscales, including medians and standard deviations, are provided in Supplementary Table S2.

### Principal component analysis

**Fig. 2:**
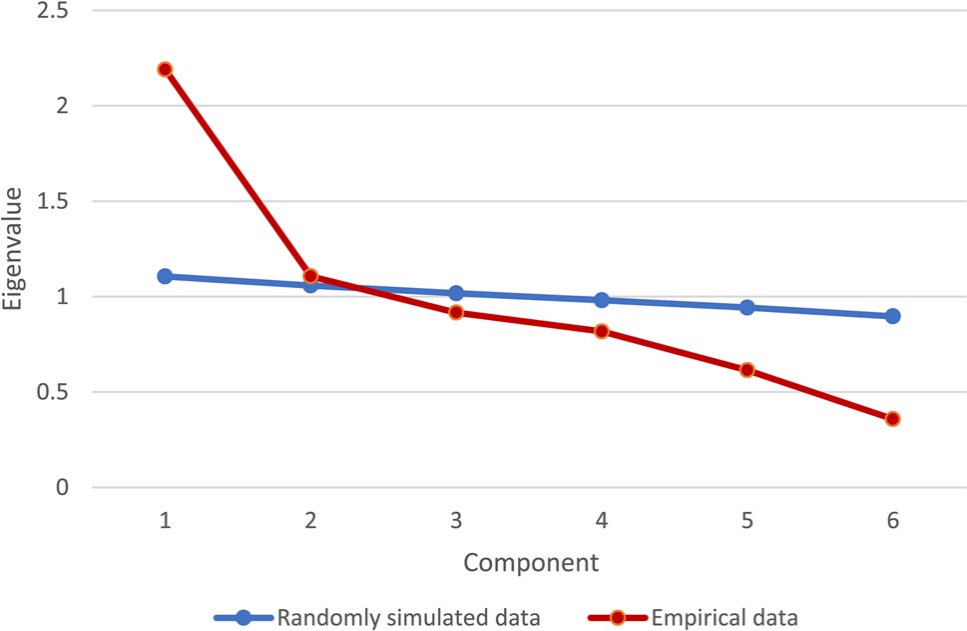
Results of the parallel analysis

For the principal component analysis, all requirements were met, including the Kaiser-Meyer-Olkin criterion (.680) and a significant result on the Bartlett test (χ² (15) = 865.95; *p* <.001). Because the scree plot results were inconclusive, we computed Horn´s parallel analysis to determine the final number of components to be extracted. The parallel analysis indicated that two components (Fig. 2) accounted for 54.9% of the total variance. After a Varimax rotation, all six items showed clear loadings (> .50) on one of the two extracted components. Items 1, 3, 5, and 6 loaded on the FC component (eigenvalue = 2.19, 36.5% of the total variance), whereas the DC component (eigenvalue = 1.12, 18.4% of the total variance) comprised Items 2 and 4. Table 2 reports the results of the final structure.

**Table 2:** Component loadings after Varimax rotation

Items	Component 1^a^ COPE 6 – FC	Component 2^a^ COPE 6 – DC
**Item 1** I've been concentrating my efforts on doing something about the situation I'm in.	**.515**	.326
**Item 3** I've been getting emotional support from others^b^	**.820**	
**Item 5** I've been getting comfort and understanding from someone.	**.839**	
**Item 6** I’ve been trying to get advice or help from other people about what to do.	**.725**	
**Item 2** I've been using alcohol or other drugs to make myself feel better.		**.692**
**Item 4** I've been giving up trying to deal with it.		**.724**

Includes only component loadings of .300 or above. *FC *functional coping, *DC *dysfunctional coping

^a^The highest loading is printed in bold

^b^The original German translation by Knoll [43] was modified slightly by the team of experts to improve comprehensibility

### Item analysis

The item difficulty of the items on the FC subscale ranged from .46 to .52 with a good mean item difficulty of .49. All items showed moderate to high discriminatory power ranging from .33 to .59 (Table 3). The difficulty of the items on the DC subscale ranged from .12 to .35 with a mean item difficulty of .24, which was considerably smaller than the mean item difficulty of the FC subscale. Discriminatory power could not be calculated because the DC subscale included only two items (Table 3).

**Table 3:** Item characteristics of the COPE 6

Subscales and Items	*M* (*SD*)	Item Difficulty	Discriminatory Power	Reliability
**FC**				**Overall scale**^**a**^
				.71
				**“If item deleted”** ^**a**^
**Item 1** I’ve been concentrating my efforts on doing something about the situation I’m in.	1.95 (1.18)	.49	.33	.74
**Item 3** I’ve been getting emotional support from others.	1.84 (1.16)	.46	.58	.60
**Item 5** I’ve been getting comfort and understanding from someone.	1.89 (1.29)	.47	.59	.59
**Item 6** I’ve been trying to get advice or help from other people about what to do.	2.09 (1.24)	.52	.50	.65
**DC**				**Overall scale** ^**b**^
				.16
				**“If item deleted”** ^**c**^
**Item 2** I’ve been using alcohol or other drugs to make myself feel better.	0.47 (0.97)	.12	n. a.	n. a.
**Item 4** I’ve been giving up trying to deal with it.	1.38 (1.13)	.35	n. a.	n. a.

*M *mean, *SD *standard deviation, *FC *functional coping, *DC *dysfunctional coping, *n. a. *not available

^a^Internal consistency based on Cronbach´s alpha (α)

^b^Split-half reliability based on the Spearman-Brown formula because the subscale included only two items [77]

^c^Calculation not available, as the subscale included only two items

### Reliability

Despite the small number of items, there was an acceptable Cronbach´s alpha of .71 for the total FC subscale. Taking out the item “I’ve been concentrating my efforts on doing something about the situation I’m in” increased Cronbach´s alpha slightly to .74 (Table 3). However, we decided not to remove this item due to the limited increase in internal consistency. In addition, by keeping all the items, the FC subscale continued to contain two items each from the underlying emotion-focused and problem-focused coping strategies and thus provided an optimal and well-balanced representation of the FC construct. The split-half reliability of the two-item DC subscale was low, represented by a Spearman-Brown coefficient of *r*_*sb*_ = .16. The reliability “If item deleted” could not be calculated because the DC subscale included only two items (Table 3).

The two subscales were uncorrelated (*r*_*p*_ = .08).

### Convergent and discriminant validity

There was a large positive correlation between the COPE 6 FC subscore and the Brief COPE FC subscore (*r*_*p*_ = .64) as well as a small positive correlation between the COPE 6 DC subscore and the Brief COPE DC subscore (*r*_*p*_ = .23). Based on these results, hypothesis 1 could be confirmed for the COPE 6 FC subscale and there was a general tendency towards the confirmation of hypothesis 1 for the DC subscale. Furthermore, there was a small positive correlation between the COPE 6 FC subscore and the Benefits of Being a Caregiver Scale score (*r*_*p*_ = .23) and a quasi-zero correlation between the COPE 6 DC subscore and the Benefits of Being a Caregiver Scale score (*r*_*p*_ = .08). There was also a small positive correlation between the short version of the Burden Scale for Family Caregivers score and the COPE 6 DC subscore (*r*_*p*_ = .17). No association was found with the COPE 6 FC subscore (*r*_*p*_ = .08). Hypothesis 2 and hypothesis 3 were supported by these results.

With the exception of a small correlation between the COPE 6 FC subscore and the Social Desirability-Gamma Short Scale *minimizing negative qualities* subscore (*r*_*s*_ = .29), the COPE 6 subscores were not associated with the Social Desirability-Gamma Short Scale subscores. Accordingly, the COPE 6 DC subscore was neither correlated with the Social Desirability-Gamma Short Scale *exaggerating positive qualities* subscore (*r*_*p*_ = .04) nor with the Social Desirability-Gamma Short Scale *minimizing negative qualities* subscore (*r*_s_ = − .05). For the COPE 6 FC subscore, there was no correlation with the Social Desirability-Gamma Short Scale *exaggerating positive qualities* subscore either (*r*_*p*_ = − .08). These findings generally supported hypothesis 4. There were no correlations between the COPE 6 FC subscore and all tested demographic factors (age: *r*_*p*_ = − .05, gender: *r*_*pb*_ = − .01, educational level: *r*_*s*_ = .02, employment status: *r*_*pb*_ = − .04). The same was found for the COPE 6 DC subscore (age: *r*_*p*_ = − .03, gender: *r*_*pb*_ = − .02, educational level: *r*_*s*_ = − .02, employment status: *r*_*pb*_ = .03). Hypothesis 5 could be fully confirmed by these findings. Table 4 presents a summary of all results.

**Table 4:** Construct validity of the COPE 6 (hypotheses 1–5)

Hypothesis	COPE 6 Subscales			
	FC		DC	
	Correlation	*p* ^a^	Correlation	*p* ^a^
**Convergent Validity**				
H1: Brief COPE^b^				
FC	*r*_*p*_ = .64	.006		
DC			*r*_*p*_ = .23	.076
H2:				
BBCS^c^	*r*_*p*_ = .23	.006	*r*_*p*_ = .08	.042
H3:				
BSFC-s^c^	*r*_*p*_ = .08	.042	*r*_*p*_ = .17	.006
**Discriminant Validity**				
H4: KSE-G^b^				
PQ+	*r*_*p*_ = − .08	.705	*r*_*p*_ = .04	.705
NQ-	*r*_*s*_ = .29	.024	*r*_*s*_ = − .05	.705
H5: Demographics^c^				
Age	*r*_*p*_ = − .05	.319	*r*_*p*_ = − .03	.537
Gender	*r*_*pb*_ = − .01	.863	*r*_*pb*_ = − .02	.698
Educational level	*r*_s_ = .02	.635	*r*_*s*_ = − .02	.635
Employment status	*r*_*pb*_ = − .04	.520	*r*_*pb*_ = .03	.557

*H* Hypothesis, *Brief COPE* Brief Coping Orientation to Problems Experienced Inventory, *FC* Functional coping, *DC* Dysfunctional coping, *BBCS* Benefits of Being a Caregiver Scale, *BSFC-s* Short version of the Burden Scale for Family Caregivers, *KSE-G* Social Desirability-Gamma Short Scale, *PQ +* Exaggerating positive qualities, *NQ-* Minimizing negative qualities, *r*_*p*_ Pearson´s correlation coefficient, *r*_*s*_ Spearman rank correlation coefficient, *r*_*pb*_ Point biserial correlation coefficient

^a ^Correction for multiple testing in the validation sample and the subsample based on Benjamini-Hochberg method

^b ^Subsample, *N* = 81

^c ^Validation sample, *N* = 961

## Discussion

The objective of this study was to validate the COPE 6 as the first theory-based extra-short adaptation of the Brief COPE. The focus was on devising an abbreviated respondent-friendly measurement tool that operationalizes the coping construct consistently and as globally as possible by considering theoretically founded broader coping dimensions. In general, the COPE 6 achieves this goal by measuring coping strategies with a total of six items that reflects the FC and DC dimensions. The FC subscale now represents a valid and reliable tool for assessing FC strategies. However, the current form of the DC subscale appears to measure two independent and extreme aspects of the DC dimension. With regard to the present DC subscale, some limitations that have implications for future research must be considered.

The COPE 6 was developed by applying a deductive, theory-based multi-step approach based on well-founded theoretical content from coping research [1, 11, 23, 26, 27, 30, 32, 60–62] and current guidelines for scale construction [63]. This approach differs from the development of other short versions of the Brief COPE that were all based primarily on statistical criteria, such as excluding items or subscales due to heterogeneous factor structures [3, 46, 93]. Thus, all previous short versions of the Brief COPE have lacked a theoretical foundation in the sense of an a priori grouping of coping strategies into dimensions [60]. To the best of our knowledge, the COPE 6 is the first a priori theory-based short form of the Brief COPE that includes fewer than 10 items.

### Factor structure

The principal component analysis revealed a two-component structure that explained 54.9% of the total variance with good component loadings ranging from .515 to .839 for the FC subscale and .692 to .724 for the DC subscale. These findings deviated from our initial theory-based expectations of a three-component solution (emotion-focused, problem-focused, and DC components), which we had hypothesized on the basis of Carver et al.’s [30] theoretical assumptions. Nevertheless, with respect to content validity, the two-component solution we found is plausible. Carver et al. [30] named the coping strategies forming the emotion-focused and problem-focused dimensions *functional* or *more useful* strategies in contrast to the *less useful*, *dysfunctional* strategies. Likewise, on an empirical level, Solberg et al. [28] reported that a dichotomous factor structure was commonly found [66, 67]. The results of these past studies on the Brief COPE support the results of the principal component analysis found in the present study for the COPE 6.

On the basis of the extracted components, we constructed the FC and DC subscales. The FC and DC subscores were not significantly associated, indicating that the two scales assess independent dimensions of the coping construct. This finding is consistent with previous research [67].

In contrast to the FC subscale, which was normally distributed, the DC subscale was right-skewed. This result is not surprising, as previous research has already shown that FC strategies are used more frequently on average than DC strategies [8, 66]. In addition, the results are in line with research that has investigated DC in the population of informal caregivers. Gottschalk et al. [94] showed that the DC strategy of risky alcohol consumption was less common in informal caregivers than in non-informal caregivers. It has been suggested that informal caregivers generally exhibit more responsible behaviors because dysfunctional behaviors carry long-term consequences that they wish to avoid due to their high level of perceived responsibility [94]. Nevertheless, there are recommendations to check for a potential social desirability bias [95]. In the current study, there is some evidence that the DC subscale from the COPE 6 was most likely not biased by social desirability because there were no meaningful associations between the DC subscale and the Social Desirability-Gamma Short Scale subscales. While the present study found no evidence of an association of the DC subscale with social desirability, future research should examine this potential bias more thoroughly.

### Reliability findings

The FC subscale had an internal consistency of .71, which can be classified as acceptable [78]. In contrast to past research, which reported good reliability values for the FC subscale of the Brief COPE ranging from .81 to .88 [8, 36, 68], the internal consistency of the COPE 6 FC subscale is lower. However, Cronbach´s alpha has to be interpreted in light of the fact that the number of items on the FC subscale is substantially lower than on the Brief COPE, thus potentially leading to the biased assumption of lower homogeneity [96, 97]. There are recommendations that, when the internal consistency is lower, the quality of a scale should be particularly scrutinized by considering other complementary validation criteria (e.g., results of a factor analysis) [63, 97]. In our study, the results of the principal component analysis were good, showing a consistent FC dimension with component loadings ranging from .515 to .839 and an eigenvalue of 2.19 for the FC component, which explained 36.5% of the total variance. In addition, the item characteristics could be classified as good as well. There were item difficulty values ranging from .46 to .52 with a mean item difficulty of .49. The discriminatory power of the items was moderate to high (.33 to .59).

In contrast to the good reliability of the FC subscale, the two-item DC subscale had a low Spearman-Brown coefficient of *r*_*sb*_ = .16 [79]. This result should be considered in light of to two aspects. First, the reliability value [77] found in this study for the DC subscale can generally be compared with estimates of the internal consistency of the Brief COPE’s DC subscale. Previous research on the Brief COPE showed that Cronbach´s alpha was inadequate for the Brief COPE’s DC subscale as well. Meyer [36] reported an insufficient Cronbach´s alpha coefficient for the DC subscale of .48. This finding is consistent with our result, with the smaller number of items accounting for the lower absolute value of the COPE 6 DC subscale. Second, the results of the reliability analysis should be supplemented by additional validation criteria. Despite the small Spearman-Brown coefficient, the principal component analysis showed good loadings of both dysfunctional items (.692 and .724) on the DC component. According to Wendt and Peterman [64], weakly correlated behaviors may still represent distinct dimensions of the same construct, particularly when one is used more frequently than the other. Considering the dysfunctional items of the COPE 6, informal caregivers scored higher on the item “I’ve been giving up trying to deal with it” (*M* = 1.38; *SD* = 1.13) than on the item “I’ve been using alcohol or other drugs to make myself feel better” (*M* = 0.47; *SD* = 0.97) on average, which explains the small Spearman-Brown coefficient. The low correlation appears inconsistent with the item content, as both capture DC strategies. Previous studies have shown that both coping strategies are associated with dysfunctional outcomes including anxiety, negative affect, general distress, physical symptoms, and depression [98].

Future studies are needed to determine whether items that appear to reflect DC strategies in content but exhibit weak statistical association represent two extreme aspects of the DC dimension, differing in intensity and activity level. Risky alcohol and drug consumption potentially reflect a higher level of intensity and a more active DC behavior than giving up to cope with the situation, which is less intensive and more passive. This is in line with research that has discussed the idea of a continuum of coping styles that are characterized by bipolar dimensions [99]. Furthermore, future research should investigate whether the item “I’ve been using alcohol or other drugs to make myself feel better” might represent two different aspects of high DC as well. Evidence supporting this assumption comes from previous research indicating that informal caregivers use benzodiazepines and related drugs more frequently and for longer periods than non-caregivers [100]. By contrast, alcohol consumption did not differ between the two populations because informal caregivers want to avoid the resulting long-term consequences of alcohol consumption [94]. Consequently, if an item includes both aspects, the more salient but less frequent aspect (alcohol consumption) may overshadow the other (medication use), biasing responses. In summary, future research should consider adding two items to the DC subscale to better capture dimensional gradations and splitting the substance use item into separate items for alcohol consumption and medication use.

### Validity findings

Convergent validity was confirmed for the COPE 6 FC subscale by a large correlation between the COPE 6 FC subscale and the Brief COPE FC subscale. As suspected, this result indicates that the COPE 6 FC subscale in its current form reflects functional coping in an adequate way. Contrary to expectations, convergent validity for the COPE 6 DC subscale could not be sufficiently confirmed. There was only a small correlation between the COPE 6 and the Brief COPE DC subscales. The weak convergent validity supports the insufficient results concerning the reliability and emphasizes the need for further development of the DC subscale. Furthermore, in accordance with our hypotheses related to convergent validity, we found a small but positive correlation between the COPE 6 FC subscore and the Benefits of Being a Caregiver Scale score as well as between the COPE 6 DC subscore and the short version of the Burden Scale for Family Caregivers score. The Benefits of Being a Caregiver Scale score was not associated with the COPE 6 DC subscore and the short version of the Burden Scale for Family Caregivers score showed no association with the COPE 6 FC subscale. These findings are in line with previous studies indicating that FC behavior is generally associated with the experience of more benefits in informal caregiving [83, 84] in contrast to dysfunctional strategies, which are more strongly correlated with informal caregivers´ perceived burden [20, 33, 52, 85–87].

The hypotheses referring to discriminant validity were largely supported. There was neither a correlation between the COPE 6 DC subscale and both Social Desirability-Gamma Short Scale subscales, nor between the COPE 6 FC subscore and the Social Desirability-Gamma Short Scale *exaggerating positive qualities* subscore. These findings align with past research results showing the Brief COPE not being associated with social desirability [88]. There was, however, a small correlation between the COPE 6 FC subscore and the Social Desirability-Gamma Short Scale *minimizing negative qualities* subscore. This result was initially surprising, but turns out to be generally in line with past research showing that some scales of the original Coping Orientation to Problems Experienced Inventory showed small but significant correlations with social desirability as well [30]. In summary, it can be concluded that the COPE 6 is relatively uncorrelated to the social desirability construct. Lastly, consistent with previous findings in coping research [e.g., 91], we confirmed that there were no associations between informal caregivers´ demographic variables (age, gender, education level, and employment status) and both COPE 6 subscores. This finding suggests that the use of dispositional coping strategies seems to be based more on specific individual factors than on general ones. This idea is supported by research that showed associations between coping strategies and personality [101] or individual attachment styles [33]. In summary, the results of the convergent and discriminant validity tests support the aforementioned argumentation of a valid and reliable FC scale and a still inadequately designed DC subscale in its current form.

### Limitations

As the first theory-based economic adaptation of the Brief COPE to assess the FC and DC strategies, the COPE 6 was validated on a large sample of 961 informal caregivers that could be considered representative of informal caregivers in Bavaria (Germany). Nevertheless, some limitations must be considered.

First, in this study, we validated the dispositional version of the COPE 6 on the homogeneous population of mostly female informal caregivers caring for elderly care receivers in Germany in order to reduce the complexity of the assessment of coping and thus sharpen the representation of the coping construct. The gender imbalance inevitably resulted from the fact that the population of informal caregivers is more strongly represented by women worldwide [90]. Consequently, the results of our study apply only to the population of German informal caregivers caring for elderly care receivers and to the dispositional version of the questionnaire. Future research is needed to investigate whether the component structure can also be found in more heterogeneous populations with a more balanced gender ratio and whether the same structure will emerge when the situational version is used.

Second, because the present validation was based on data that were collected for a different primary focus, complete information for the analysis of convergent (Brief COPE) and discriminant (Social Desirability-Gamma Short Scale) validity was missing. Thus, we had to collect this missing information in a subsample. To ensure the comparability of the two samples, the subsample comprised individuals from the validation sample. In addition, we assessed dispositional coping, which is assumed to be relatively stable against contextual factors, such as time and various situations [102].

Third, there is an imbalance between the number of items on the FC subscale and the number on the DC subscale. This imbalance arose organically over the course of the validation process. We first developed the scale under the theoretically supported assumption of the existence of the three-factor structure of problem-focused, emotion-focused, and DC in the Brief COPE. This structure had already been theoretically proposed by Carver et al. [30] and was also supported by multiple previous studies [28], particularly in the population of informal caregivers we studied [11, 22, 33]. However, our study showed contrasting empirical results in the form of a two-component structure. The resulting FC subscale included four items that reflected the emotion-focused and problem-focused scales. Consequently, the DC subscale comprised only two items. The psychometric properties of two-item scales can be interpreted only to a limited extent. These aspects must be considered when assessing the present results. Further research is needed to test whether the integration of two additional items into the DC subscale can assess the gradations of the dimension in a more precise and balanced manner and can potentially increase the currently variance of 18.4% explained by the DC subscale.

Finally, for reasons of economy, this study focused only on the core psychometric criteria of the COPE 6. Consequently, it was not possible to conduct an exhaustive examination of its criterion validity by testing the association of the COPE 6 with external criteria. Given the practical relevance of the associations of the FC and DC dimensions with constructs such as physical health, mental health, and well-being [22, 103–105], future research should be devoted to examining these associations in order to provide an assessment of its potential clinical benefits.

## Conclusion

This study is the first to validate an a priori theory-based extra-short form of the Brief COPE. The COPE 6 is a time-efficient and respondent-friendly measurement tool for the assessment of FC and DC strategies. Nevertheless, there is an imbalance between the FC and the DC subscales. The FC subscale represents a valid and reliable tool for collecting data on FC strategies. By contrast, the current two-item version of the DC subscale appears to measure two independent extreme aspects of DC. Further research is needed to develop this subscale further, for example by including two or more additional items to capture the gradations of coping with greater precision and balance. Altogether, the current version of the COPE 6 demonstrates considerable potential to measure FC and DC in a valid and more economical way than the Brief COPE. Further research is needed to enhance the DC subscale even more comprehensively and to implement an improved version in other populations.

## Supplementary Information

Supplementary Material 1: Figure S1. Flow Chart. File contains the data collection process

Supplementary Material 2: Figure S2. Distributions of the COPE 6 subscores FC (a) and DC (b). File contains the distributions of both subscores.

Supplementary Material 3: Table S1. Characteristics of the subsample (*N *= 81). File contains the subsample characteristics.

Supplementary Material 4: Table S2. Descriptive statistics for all (sub)scales. File contains the descriptive statistics (median, standard deviation) for all (subs)scales of the total sample and the subsample.

Supplementary Material 5: Appendix A. COPE 6 – English version. File contains the items and the response format of the COPE 6 (English version.

Supplementary Material 6: Appendix B. COPE 6 – German version. File contains the items and the response format of the COPE 6 (German version).

## Data Availability

The data sets used and analyzed during the current study are available from the corresponding author on reasonable request.

## Abbreviations

Brief COPE: Brief Coping Orientation to Problems Experienced Inventory
DC: Dysfunctional coping
FC: Functional coping
