# Supplementary material for: Coping strategies in challenging situations among informal caregivers: validation of the newly developed six-item German short version of the Brief COPE Inventory (COPE 6)
Source: BMC Psychol. 2025 Dec 13;14:118. doi: 10.1186/s40359-025-03815-5 (PMC12849300; doi:10.1186/s40359-025-03815-5)
Supplement: Supplementary file 1 — Supplementary Material 1: Figure S1. Flow Chart. File contains the data collection process [file 40359_2025_3815_MOESM1_ESM.docx]

**Supplementary Figure S1** Flow Chart

**Questionnaire distributed by MD to ICGs** *N* = 5,000

Questionnaires not returned *n* = 3,916

**Assessed for eligibility** *n* = 1,084

**Excluded** (*n* = 2)

Total missing values > 50% *n* = 2

*N* = 1,082

**Excluded** (*n* = 906)

No consent to be resurveyed *n* = 773

Other reasons *n* = 67

CR passed away *n* = 51

No further contact desired *n* = 13

ICG illness *n* = 2

**Subsequent distribution** **to a subsample** *n* = 176

Total recruited – *N* = 81

**Subsample used to obtain missing variables for entire examination of construct validity**^2^

Questionnaires not returned *n* = 70

**Assessed for eligibility** *n* = 106

**Excluded** (*n* = 121)

CRs < 65 years *n* = 121

Total recruited – *N* = 961

**Validation sample**^2^

**Excluded** (*n* = 25)

CRs younger than 65 *n* = 16

Total missing values > 50%^1^ *n* = 7

Implausible response pattern^1^ *n* = 2

*Note.* The path followed for data collection in the validation sample is shown in bold lined boxes, the path followed in the subsample is shown in thin lined boxes. MD = Medical Service of the Bavarian Health Insurance, ICG(s) = informal caregiver(s), CR(s) = care receiver(s). ^1^ In the Brief COPE and/or the Social Desirability-Gamma Short Scale; ^2^ The majority of the analyses were performed in the validation sample; two missing variables to test construct validity entirely (Brief COPE, Social Desirability-Gamma Short Scale) had to be collected in a subsample of the main “Benefits of being a caregiver” sample.
