# Supplementary material for: Coping strategies in challenging situations among informal caregivers: validation of the newly developed six-item German short version of the Brief COPE Inventory (COPE 6)
Source: BMC Psychol. 2025 Dec 13;14:118. doi: 10.1186/s40359-025-03815-5 (PMC12849300; doi:10.1186/s40359-025-03815-5)
Supplement: Supplementary file 2 — Supplementary Material 2: Figure S2. Distributions of the COPE 6 subscores FC (a) and DC (b). File contains the distributions of both subscores. [file 40359_2025_3815_MOESM2_ESM.docx]

**Supplementary Figure S2** Distributions of the COPE 6 subscores FC (a) and DC (b)


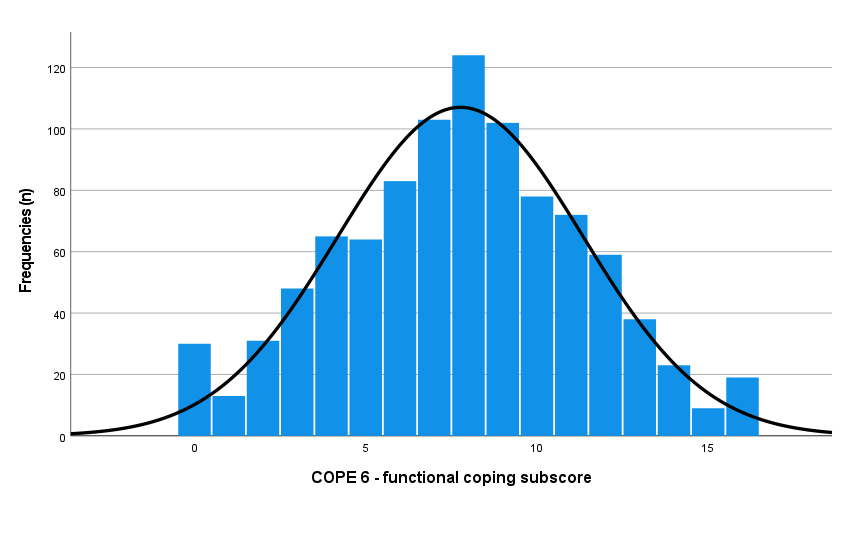


**a**

**
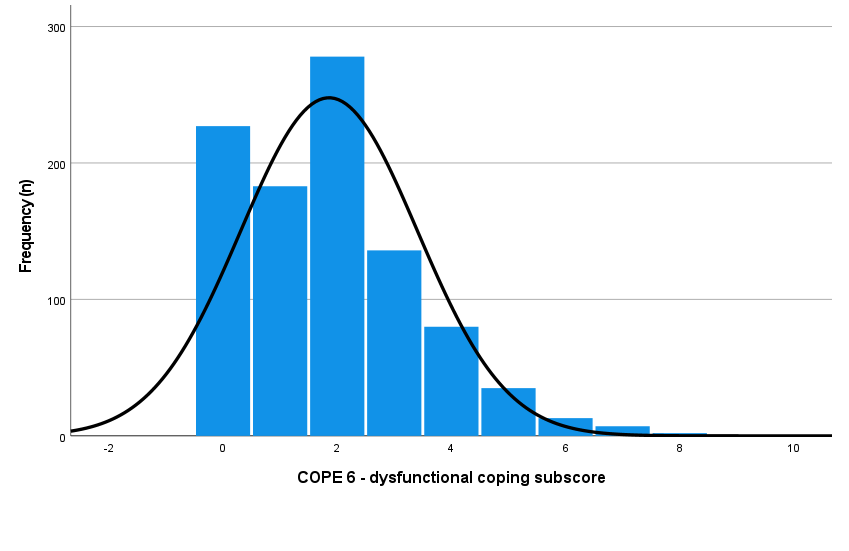
**

**b**

*Note.* Higher scores on the *COPE 6 - functional coping (FC) subscale* indicate the use of more functional coping strategies (a, Range: 0–16). Higher scores on the *COPE 6 - dysfunctional coping (DC) subscale* indicate the use of more dysfunctional coping strategies (b, Range: 0–8).
