# Supplementary material for: Coping strategies in challenging situations among informal caregivers: validation of the newly developed six-item German short version of the Brief COPE Inventory (COPE 6)
Source: BMC Psychol. 2025 Dec 13;14:118. doi: 10.1186/s40359-025-03815-5 (PMC12849300; doi:10.1186/s40359-025-03815-5)
Supplement: Supplementary file 3 — Supplementary Material 3: Table S1. Characteristics of the subsample (N = 81). File contains the subsample characteristics. [file 40359_2025_3815_MOESM3_ESM.docx]

**Supplementary Table S1** Characteristics of the subsample (*N* = 81)

| **Characteristics** | ***n* (%)** | ***M* (*SD*)** |
| --- | --- | --- |
| **Informal caregiver** |  |  |
| Age (years) |  | 65.11 (12.36) |
| Gender (female) | 62 (76.5) |  |
| Highest education level |  |  |
| Lower secondary school | 30 (37.0) |  |
| Middle school | 33 (40.7) |  |
| Advanced school-leaving  examination | 10 (12.4) |  |
| University degree | 8 (9.9) |  |
| Gainfully employed (yes) | 31 (38.3) |  |

*Note**.* *n*/*N* = sample size; *M* = mean; *SD* = standard deviation.
