# Supplementary material for: Coping strategies in challenging situations among informal caregivers: validation of the newly developed six-item German short version of the Brief COPE Inventory (COPE 6)
Source: BMC Psychol. 2025 Dec 13;14:118. doi: 10.1186/s40359-025-03815-5 (PMC12849300; doi:10.1186/s40359-025-03815-5)
Supplement: Supplementary file 4 — Supplementary Material 4: Table S2. Descriptive statistics for all (sub)scales. File contains the descriptive statistics (median, standard deviation) for all (subs)scales of the total sample and the subsample. [file 40359_2025_3815_MOESM4_ESM.docx]

**Supplementary Table S2** Descriptive statistics for all (sub)scales

| **(Sub)Scale** | **Sample**  (*N* = 961) | **Subsample**  (*N* = 81) |
| --- | --- | --- |
|  | ***M* (*SD*** | ***M* (*SD*** |
| COPE 6 – FC | 7.77 (3.58) | 6.81 (3.78) |
| COPE 6 – DC | 1.86 (1.55) | 2.00 (1.46) |
| Brief COPE – FC |  | 22.25 (7.23) |
| Brief COPE – DC |  | 7.73 (3.35) |
| KSE-G – PQ+ |  | 2.91 (0.67) |
| KSE-G – NQ- |  | 0.37 (0.54) |
| BBCS | 26.14 (12.37) |  |
| BSFC-s | 16.71 (7.49) |  |

*Note**.* *N* = sample size; *M* = mean; *SD* = standard deviation; COPE 6 – FC = functional coping subscale from the COPE 6 (Range 0–16); COPE 6 – DC = dysfunctional coping subscale from the COPE 6 (Range 0–8); Brief COPE – FC = functional coping subscale from the Brief Coping Orientation to Problems Experienced Inventory (Range 0–51); Brief COPE - DC = dysfunctional coping subscale from the Brief Coping Orientation to Problems Experienced Inventory (Range 0–33); KSE-G – PQ+ = Social Desirability-Gamma Short Scale – subscale Exaggerating Positive Qualities (Range 0–4); KSE-G – NQ- = Social Desirability-Gamma Short Scale – subscale Minimizing Negative Qualities (Range 0–4); BBCS = Benefits of Being a Caregiver Scale (Range 0-56); BSFC-s = Short version of the Burden Scale for Family Caregivers (Range 0-30).
