# Supplementary material for: Coping strategies in challenging situations among informal caregivers: validation of the newly developed six-item German short version of the Brief COPE Inventory (COPE 6)
Source: BMC Psychol. 2025 Dec 13;14:118. doi: 10.1186/s40359-025-03815-5 (PMC12849300; doi:10.1186/s40359-025-03815-5)
Supplement: Supplementary file 5 — Supplementary Material 5: Appendix A. COPE 6 – English version. File contains the items and the response format of the COPE 6 (English version. [file 40359_2025_3815_MOESM5_ESM.docx]

**Appendix A** COPE 6 – English version

| **Item Description** | **Strongly agree** | **Agree** | **Partly**  **agree** | **Disagree** | **Strongly disagree** |
| --- | --- | --- | --- | --- | --- |
| I've been concentrating my  efforts on doing something  about the situation I'm in^a^ | **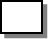** | **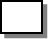** | **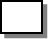** | **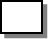** | **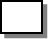** |
| I've been using alcohol or  other drugs to make myself  feel better^b^ | **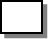** | **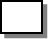** | **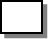** | **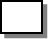** | **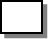** |
| I've been getting emotional  support from others^c^ | **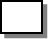** | **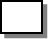** | **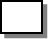** | **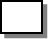** | **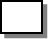** |
| I've been giving up trying to  deal with it^d^ | **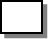** | **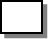** | **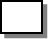** | **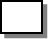** | **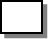** |
| I've been getting comfort  and understanding from  someone^c^ | **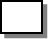** | **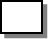** | **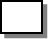** | **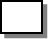** | **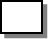** |
| I’ve been trying to get  advice or help from other  people about what to do^e^ | **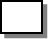** | **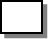** | **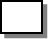** | **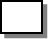** | **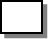** |

*Note.* Items were taken from the original Brief COPE subscales [1].

^a^ Active coping

^b^ Substance use

^c^ Using emotional support

^d^ Behavioral disengagement

^e^ Using instrumental support

**Reference**

1. Carver CS. You want to measure coping but your protocol's too long: consider the Brief COPE. Int J Behav Med. 1997; 4(1):92-100.
