# Supplementary material for: Coping strategies in challenging situations among informal caregivers: validation of the newly developed six-item German short version of the Brief COPE Inventory (COPE 6)
Source: BMC Psychol. 2025 Dec 13;14:118. doi: 10.1186/s40359-025-03815-5 (PMC12849300; doi:10.1186/s40359-025-03815-5)
Supplement: Supplementary file 6 — Supplementary Material 6: Appendix B. COPE 6 – German version. File contains the items and the response format of the COPE 6 (German version). [file 40359_2025_3815_MOESM6_ESM.docx]

**Appendix B** COPE 6 – German version

| Item description | Trifft voll zu | Trifft eher zu | Trifft teilweise zu | Trifft eher nicht zu | Trifft gar nicht zu |
| --- | --- | --- | --- | --- | --- |
| Ich habe mich darauf konzentriert, etwas an meiner  Situation zu verändern. | 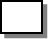 | 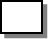 | 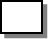 | 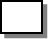 | 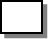 |
| Ich habe Alkohol oder andere  Mittel zu mir genommen, um  mich besser zu fühlen. | 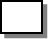 | 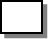 | 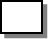 | 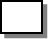 | 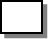 |
| Ich habe Aufmunterung von  anderen erhalten.^a^ | 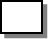 | 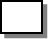 | 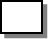 | 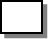 | 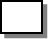 |
| Ich habe es aufgegeben,  mich damit zu beschäftigen. | 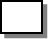 | 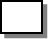 | 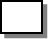 | 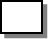 | 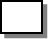 |
| Jemand hat mich getröstet  und mir Verständnis  entgegengebracht. | 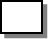 | 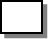 | 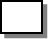 | 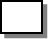 | 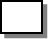 |
| Ich habe versucht, von  anderen Menschen Rat  oder Hilfe einzuholen. | 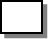 | 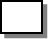 | 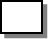 | 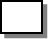 | 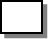 |

*Note.* ^a^The original wording was adapted to improve content validity by resolving linguistic ambiguity.
